# Supplementary figures and images for: Investigating the native functions of [NiFe]-CODH through genomic context analysis
Source: eLife. 2026 Apr 7;15:RP108780. doi: 10.7554/eLife.108780 (PMC13056359; doi:10.7554/eLife.108780)

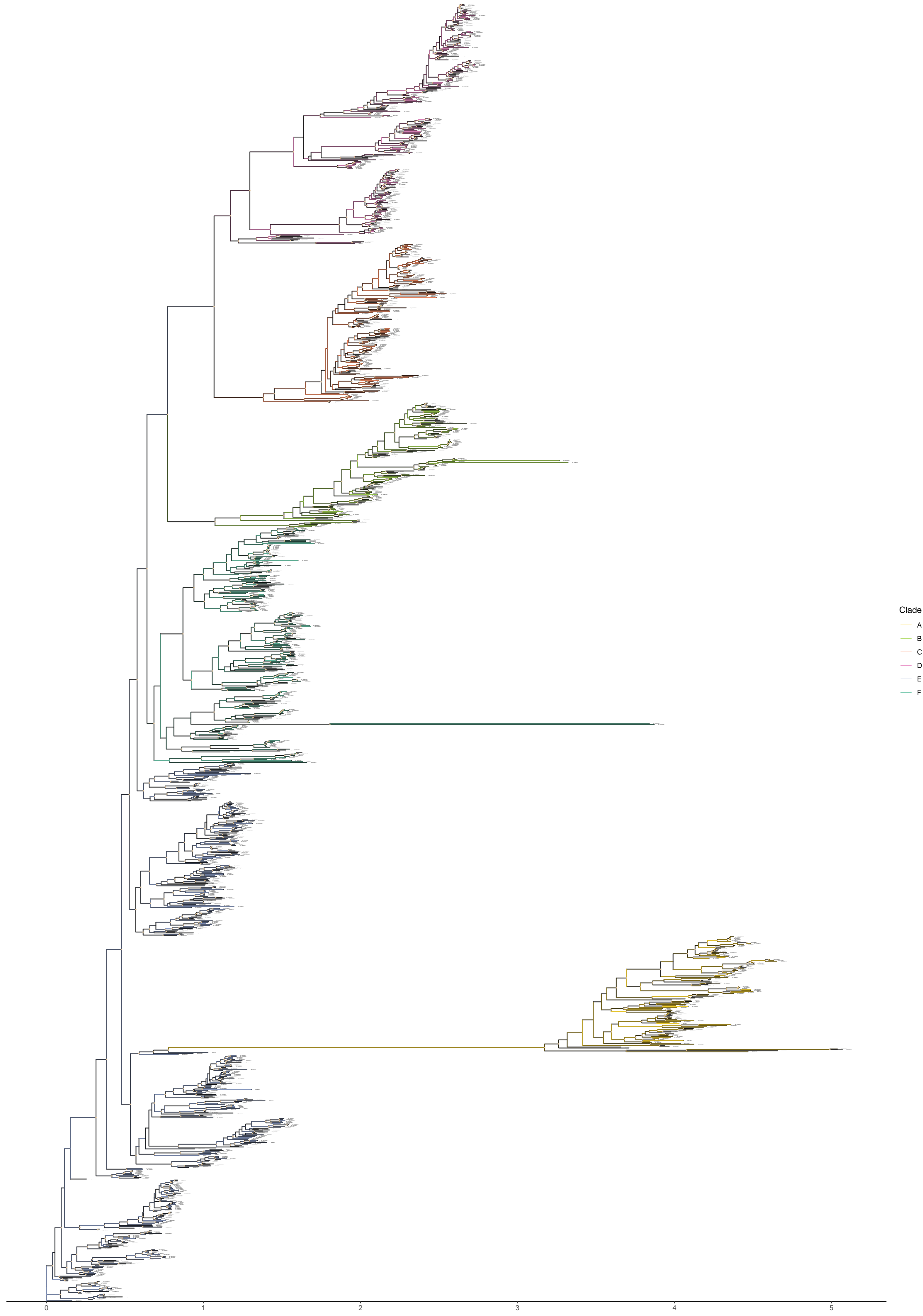

Supplement: Supplementary file 6. — Nodes supported with bootstrap values above 0.90 are marked with an orange dot. Ultrafast bootstrapping was performed using 1000 resamplings. Unrooted. [file elife-108780-supp6.zip › SupplementaryFile6.pdf]

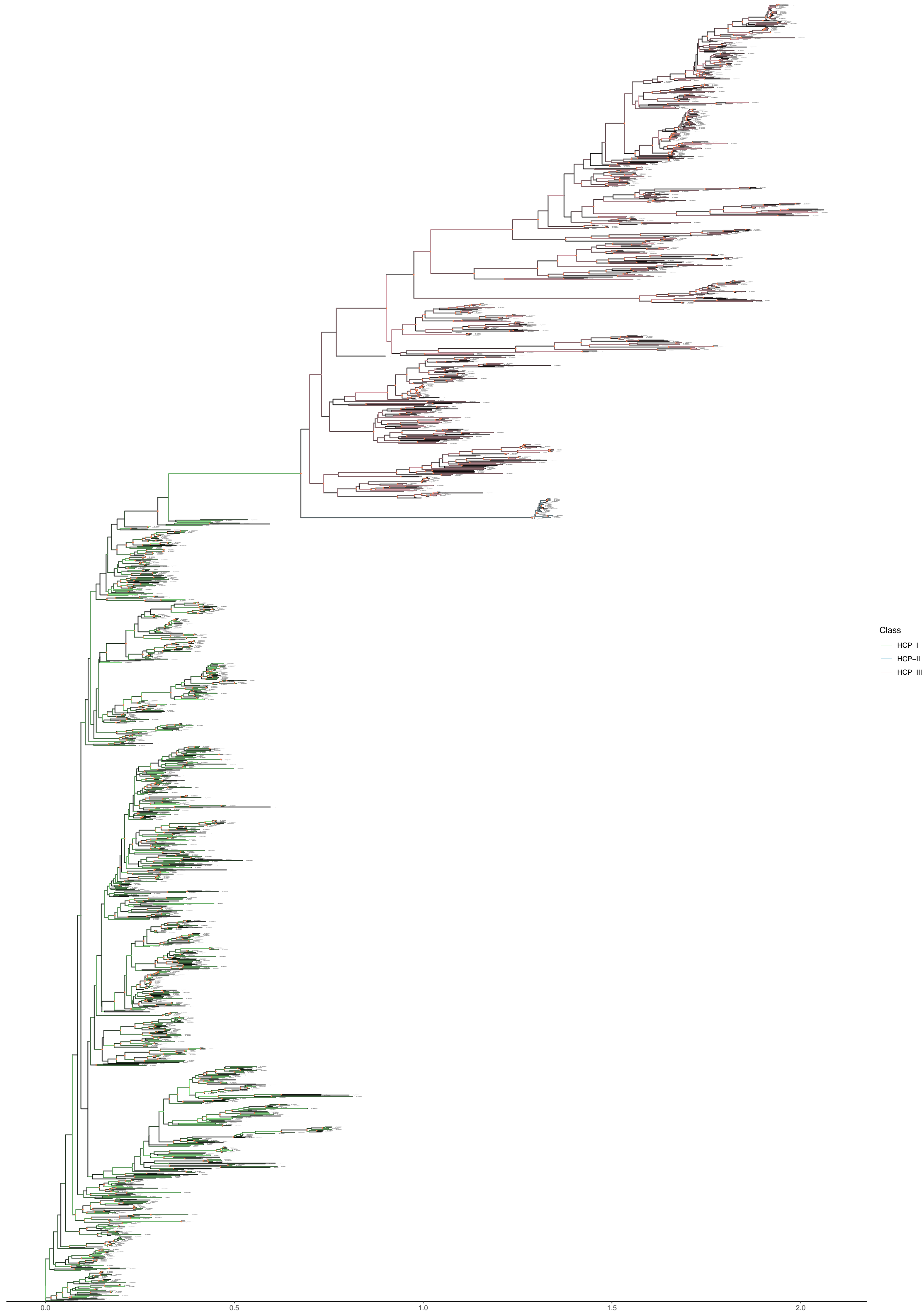

Supplement: Supplementary file 8. — Nodes supported with bootstrap values above 0.90 are marked with an orange dot. Ultrafast bootstrapping was performed using 1000 resamplings. Unrooted. [file elife-108780-supp8.zip › SupplementaryFile8.pdf]

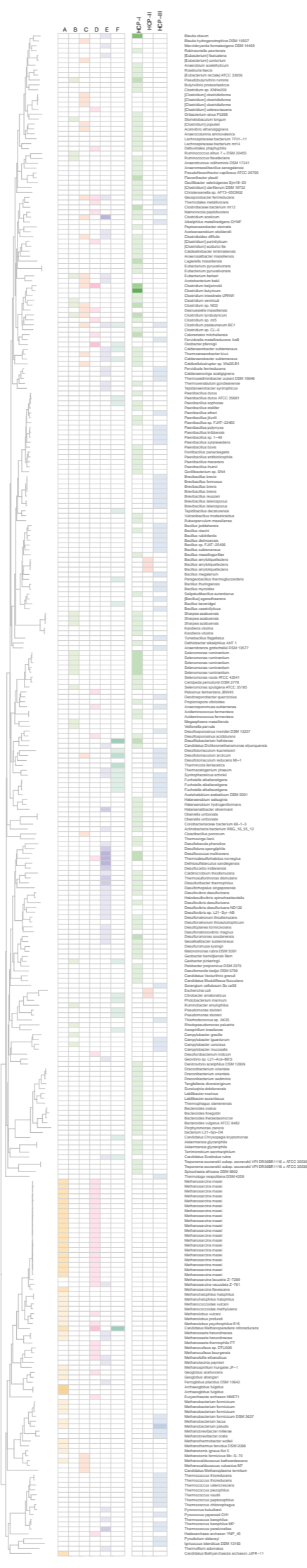

Supplement: Supplementary file 11. [file elife-108780-supp11.zip › SupplementaryFile11.pdf]
