# Supplementary material for: Investigating the native functions of [NiFe]-CODH through genomic context analysis
Source: eLife. 2026 Apr 7;15:RP108780. doi: 10.7554/eLife.108780 (PMC13056359; doi:10.7554/eLife.108780)
Supplement: Supplementary file 5. — Nodes supported with bootstrap values above 0.90 are marked with an orange dot. Ultrafast bootstrapping was performed using 1000 resamplings. Rooted using Desulfovibrio desulfuricans’ HCP (MBE6442607.1). [file SupplementaryFile5.pdf › SupplementaryFile5.pdf]

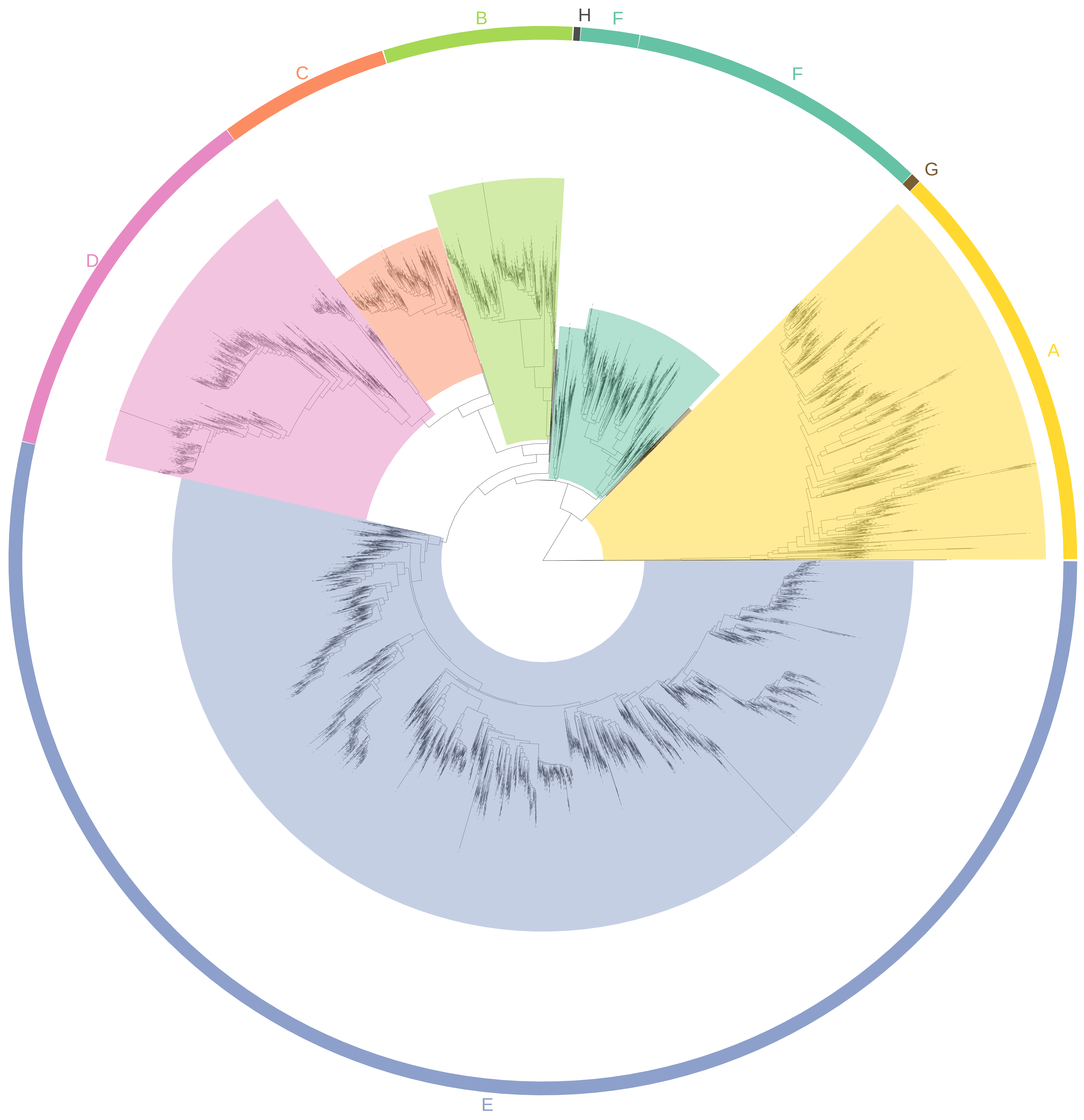
B
H
F
C
F
G
D
A
E
