# Supplementary material for: Investigating the native functions of [NiFe]-CODH through genomic context analysis
Source: eLife. 2026 Apr 7;15:RP108780. doi: 10.7554/eLife.108780 (PMC13056359; doi:10.7554/eLife.108780)
Supplement: Supplementary file 7. [file SupplementaryFile7.pdf › SupplementaryFile7.pdf]

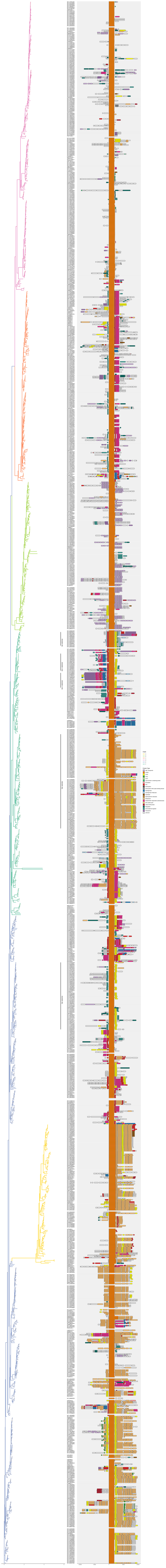

Clade
A
B
C
D
E
F
Protein Type
ABC Transporter
CODH
CooC
CooJ
CooT
cupin domain−containing protein
Ferredoxin
FeS
hemerythrin
Hemerythrin HHE cation binding domain
Hydrogenase
Hydrogenase−Maturase
Hydrolase
hydroxylamine reductase
Metabolism
NAD(P)/FAD−dependent oxidoreductase
One carbon pool
radical SAM protein
Regulation
transcriptional regulator
Transporter
unknown
