# Supplementary material for: Investigating the native functions of [NiFe]-CODH through genomic context analysis
Source: eLife. 2026 Apr 7;15:RP108780. doi: 10.7554/eLife.108780 (PMC13056359; doi:10.7554/eLife.108780)

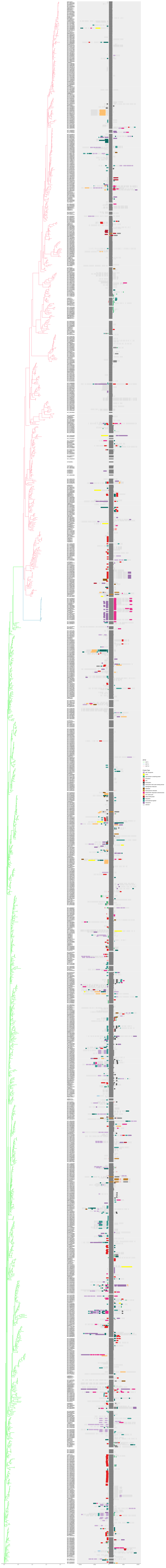

Supplement: Supplementary file 9. [file SupplementaryFile9.pdf › SupplementaryFile9.pdf]
