# Supplementary material for: Investigating the native functions of [NiFe]-CODH through genomic context analysis
Source: eLife. 2026 Apr 7;15:RP108780. doi: 10.7554/eLife.108780 (PMC13056359; doi:10.7554/eLife.108780)
Supplement: Supplementary file 10. [file SupplementaryFile10.pdf › SupplementaryFile10.pdf]

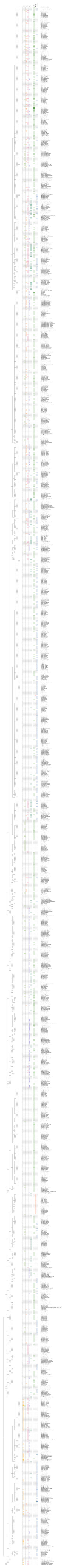
A B C D E F
HCP−I
HCP−II
HCP−III
